# Adhesion, Thermal Conductivity, and Impact on Indoor Air Quality of Plasters Incorporating Rice Husks

**DOI:** 10.3390/ma19030590

**Published:** 2026-02-03

**Authors:** Irina Popa, Cristian Petcu, Vasilica Vasile, Andreea Hegyi

**Affiliations:** 1 NIRD URBAN-INCERC Bucharest Branch, 266 Șoseaua Pantelimon, 021652 Bucharest, Romania; cristian.petcu@incd.ro (C.P.); vasile@incd.ro (V.V.); 2 NIRD URBAN-INCERC Cluj-Napoca Branch, 117 Calea Florești, 400524 Cluj-Napoca, Romania; andreea.hegyi@incerc-cluj.ro

**Keywords:** agro-industrial by-products, waste, circular economy, hybrid recipes, TVOC emissions, sustainable building materials

## Abstract

The global population growth and the demand for agricultural food products have generated a significant volume of agro-industrial by-products which, inadequately managed, affect the quality of the environment. The construction industry, a large consumer of raw materials and energy, constitutes an important source of waste and greenhouse gas emissions. In this context, the circular economy provides the right framework for the valorization of such natural materials, allowing us to obtain innovative sustainable building materials. The paper presents experimental research that led to the development of twelve plasters incorporating rice husks that were characterized by means of thickness (2.71–6.26 mm, when applied on concrete, and 4.20–10.29 mm, when applied on plasterboards), adhesion to the concrete surface (0.18–0.65 N/mm^2^), thermal conductivity (0.072–0.083 W/m·K), and impact on indoor air quality, in terms of total volatile organic compounds (TVOCs) emissions (3272–9470 µg/m^3^). The determined levels of the emissions suggest the possibility that by extending the monitoring for at least seven days after application, the information is more relevant. The findings confirmed that using the rice husks for the obtaining of such plasters represents a possible direction of valorization in construction; additional research is necessary for a more precise delineation of the characteristics of these products.

## 1. Introduction

One of the major factors influencing the overall environmental impact of the construction sector are construction materials, due to their production, transport, use, and lifespan [1]. To mitigate this problem, some strategies have been studied and implemented, such as using local materials, obtaining new, ecological materials, encouraging circular economy practices, developing new construction technologies, etc. Agriculture and related industries, such as the food and textile industries, generate diverse agro-industrial by-products and waste, whose integration into the circular economy still requires further studies and experimental research [1,2].

On the other hand, agriculture is one of the main sectors generating impressive amounts of waste [3,4,5] which has led to concern among the public and the scientific community. Straw, stalks, fibrous residues, husks, and cobs represent a significant part of agricultural secondary products that can be exploited with different techniques, such as thermochemical and biological processes, and can be transformed into fuels and value-added materials [3,5,6].

Such organic raw materials can be used much more responsibly, with increased efficiency and lower processing costs, while reducing waste, reusing, and maximizing the value of resources can generate innovative products, promoting sustainable development and generating ecological and socio-economic benefits [7]. By valorizing agricultural waste, farmers and agro-industries can contribute to sustainable practices, resource efficiency, and the circular economy [3]. On the one hand, the circular economy aims to minimize waste generated by optimizing its use and, on the other hand, increase the economic value of the resulting products, trying to extend the maximum lifespan of their components [7].

Agricultural by-products and the concept of circular economy are interconnected, both being essential for promoting sustainability in the agricultural sector [3]. Natural agro-industrial by-products represent a promising solution to these challenges. For example, straw, rice husks, sunflower husks, hemp, and other fibers of plant or animal origin are often underutilized waste products from agricultural and industrial processes. By harnessing the potential of these materials for sustainable construction applications, it is possible to avoid sending such natural resources to landfills, reduce emissions associated with traditional construction materials, and contribute to the circular economy. Current research trends in the field of sustainable construction emphasize the use of this type of natural materials in the development of environmentally friendly construction products. Comparing to the use of traditional materials, natural agro-industrial by-products offer numerous advantages, the main ones being reduced environmental impact, lower energy demand, cost-effectiveness, wide availability, favorable thermal and mechanical properties [2,8,9,10,11], acoustic performances [12,13,14], low cost, and biodegradability [15]. The integration of traditional and sustainable building materials for thermal insulation has been recognized as a viable strategy for reducing the environmental footprint of construction activities [16].

Researchers are focusing on studies that aim to minimize the cost of raw materials and improve the quality of the obtained product, objectives that can also be achieved by valorizing by-products and waste from agriculture and industry [17]. Also, the awareness of the negative impact of petroleum-based plastics, as well as the depletion of petroleum resources, has led to a change in the use of environmentally friendly materials, thus helping to generate and preserve a pollution-free environment [12,18]. Therefore, it was essential that the construction industry also move towards a more environmentally friendly approach to the various specific stages, such as design, execution, etc. [18].

In the range of the natural agro-industrial by-products, one of the representative elements is rice husks (RHs)—the hard, protective shell of the rice grain that results as a by-product in the rice winnowing process and which often ends up abandoned in the vicinity of harvesting areas, with an average of 2 million tons accumulated per year, thus contributing to environmental damage [4,19,20,21].

Due to its compositional peculiarities, this natural by-product demonstrates a diversity of possibilities for use as such. For example, in different cultivation areas, this natural agricultural by-product is either treated as waste, often buried in the ground, as a natural fertilizer [22] or as animal feed [23]; through combustion, it is used as a fuel, due to its high calorific value, to generate heat and thermal energy in industrial installations or electricity, even though the combustion process represents a danger to both human health and the environment [24,25,26].

RH is rich in cellulose, hemicelluloses, lignin, and silica [27], making it suitable for various applications in the construction industry, often being incorporated into various composite materials to provide cementitious properties, thermal insulation, acoustic performance, and/or fire resistance. Applications of this type include:

- cementitious materials—rice husk ash (RHA), a by-product of the combustion of RH, which can be used as an additional material in concrete production, improving the strength and durability of concrete while reducing its environmental impact [28];
- lightweight mortar containing agro-wastes—partial replacement of ordinary Portland cement with 15% Bagasse Ash (BA) and 15% RHA resulted in a decrease in thermal conductivity of up to 31% compared to the original mortar. Also, additions of up to both 15% RHA and 15% BA resulted in mortars with appropriate compressive strengths [29];
- addition to improve the physical and mechanical properties of bricks used as masonry elements—with the increase in the amount of RH in the ceramic masonry elements, the thermal conductivity decreased, reaching from 0.50 W/m·K to 0.27 W/m·K for a mass content of 6% embedded rice husks [30];
- bricks and blocks—RH can be incorporated as a lightweight aggregate in bricks and blocks, improving their thermal properties and reducing their weight [31];
- thermal and acoustic insulation materials—RH is used as a filler or binder in the production of insulation materials, such as panels, boards, or filler insulation [32,33,34,35];
- composite materials based on natural rubber and RHA for the development of vibration isolation supports [36];
- biocomposites—by combining with a matrix of natural or synthetic polymers, RH can be used as a reinforcing material in biocomposites, on the basis of which works such as cladding, interior or terrace finishes, wall panels, ceiling tiles, and furniture components can be carried out [15,24,37,38].

The interest of the international scientific community regarding the superior valorization of plant materials in construction is also increasingly evident with regard to another category of products, namely plasters or plaster-type coatings. Natural materials such as rice husks or hazelnut shells, corn cob granular cork from recycled bottle caps, straw fibers, etc., are used as ingredients, by partially replacing the traditional base material, resulting in a reduction in density and an improvement in the thermal insulation performance of the initial construction products. One such example is the case where by incorporating 20% rice husks into the initial matrix of a common plaster, there is a decrease of 188.80 kg/m^3^ in density and an improvement of 0.27 W/m·K in thermal conductivity [39]. Similar approaches using other agro-industrial by-products, such as sunflower seed husks, have demonstrated comparable thermal insulation improvements in coating applications [40], confirming the broader viability of agricultural waste valorization for building material development.

Recent experimental studies have further validated the thermal insulation potential of rice husk-based materials. A 2023 investigation on earthen plaster stabilized with fermented rice husk demonstrated thermal conductivity reductions from 0.87 to 0.05 W/m·K, with rice husk contents ranging from 0 to 67% by volume, with 33% rice husk providing optimal engineering performance [41]. Similarly, rice husk-based thermal insulators tested according to the methodology described in [42], thermal conductivity values of 0.073 W/m·K were obtained. Optimized formulations using pulping methods with NaOH concentration control reached exceptionally low thermal conductivity values of 0.037–0.042 W/m·K [43], while recent work on flood sediment adobe bricks enhanced with rice husk demonstrated both improved compressive strength (13.6–82.5% increase) and reduced thermal conductivity (0.200–0.278 W/m·K) [44]. These findings confirm that rice husk incorporation consistently improves thermal insulation performance across different binder systems and preparation methods.

Construction materials such as finishing/protection—paints, varnishes, or plasters—together with furniture, occupant activities (cooking, smoking, cleaning, etc.), or household cleaning products [45] represent sources for indoor pollution. Climate change and increased urbanization are two of the main factors leading to changes in the indoor environment of homes [46], with the indoor environment contributing substantially to overall exposure, as people spend approximately 90% of their time indoors [47,48,49,50,51], of which approximately 70% in homes [46]. One of the main categories of indoor pollutants are volatile organic compounds (VOCs) [52,53,54]. This type of pollutant is characterized by high volatility, severe toxicity, and poor degradability, properties which can affect human health and environment. Adverse effects on human health caused by exposure to indoor VOC concentrations include dizziness, irritation [54,55], fatigue [55], respiratory diseases [55,56], and symptoms related to sick-building syndrome [9,10,57,58].

In this context, among the concerns of researchers at international level regarding the impact on the environment of different types of products, including plasters obtained by incorporating natural materials, the determination of TVOC and formaldehyde emissions can be mentioned [59]. Recent research has highlighted that environmental factors such as temperature, relative humidity, and air exchange rate significantly influence VOC emission rates from building materials, with emissions typically peaking rapidly upon installation before declining and stabilizing within predictable ranges [60]. Furthermore, long-term monitoring studies extending up to 431 days have demonstrated that while initial VOC concentrations diminish exponentially with material aging, diffusion coefficients and partition coefficients remain relatively stable [61], suggesting that proper ventilation strategies remain essential for maintaining WHO indoor air quality standards. Comparative studies on bio-based materials have shown that optimization of natural binders can reduce TVOC emissions by 70–80% compared to conventional formulations (from baseline levels of ~3775 µg/m^3^ to ~880 µg/m^3^) [62].

Taking all these aspects into account, the main goal of this experimental research is to identify a new possible direction for the use of rice husks in construction. The experimental study aims to develop plasters by incorporating RH focusing on two basic innovative hypotheses, as follows:

1. Plasters of this type are mainly composed of two basic materials, RH and any synthetic binder available on the construction materials market as an interior or exterior finish, provided that it has a certain content of non-volatile matter. Depending on the recipe, the composition may also include a synthetic adhesive.
2. Plasters incorporating rice husks (PRHs) can be made and applied even by individual users, provided that no prior preparation of the RH is required and the mixing and homogenization of the components can be performed manually or with usual technical equipment. The biomaterials are able to be mixed and homogenized mechanically or manually, resulting in finishing plasters with good adhesion to the surface—concrete substrate of at least 0.3 N/mm^2^—according to Standard SR EN 15824 [63].

The study covers the following aspects:

- The main purpose is to explore a new potential direction for utilizing rice husks in construction by producing plasters. The element of originality lies in obtaining plasters by incorporating rice husks into various binders and adhesives, unlike most of the studies reported in the specialized literature, which aimed at partially replacing the binder in a traditional product with rice husks.
- The paper presents the materials that were used, the compositional aspects of the obtained plasters, and also the criteria and the methods applied for the interdisciplinary study of the resulting products. The following quantitative indicators were determined for characterizing the plasters: the thickness and adhesion to the concrete surfaces, the thermal insulation properties, and the impact on indoor air quality by TVOC emissions monitoring. The specific test samples, the challenges encountered during laboratory work, but above all, the good and very good experimental results obtained are presented.
- The potential of this method of capitalizing rice husks in construction has been documented and supported, as well as specified; the future research directions are necessary to fulfill in order to obtain a more detailed assessment of the areas of construction use for this type of plaster.

## 2. Materials and Methods

The laboratory research presented in the paper led to obtaining twelve variants of sustainable plasters. These products were tested by means of thickness, adhesion to concrete, thermal conductivity, and impact on indoor air quality, in order to confirm that the use of RH for obtaining such plasters could represent a direction of valorization with an encouraging potential.

### 2.1. Materials Characterization

**Figure 1:**
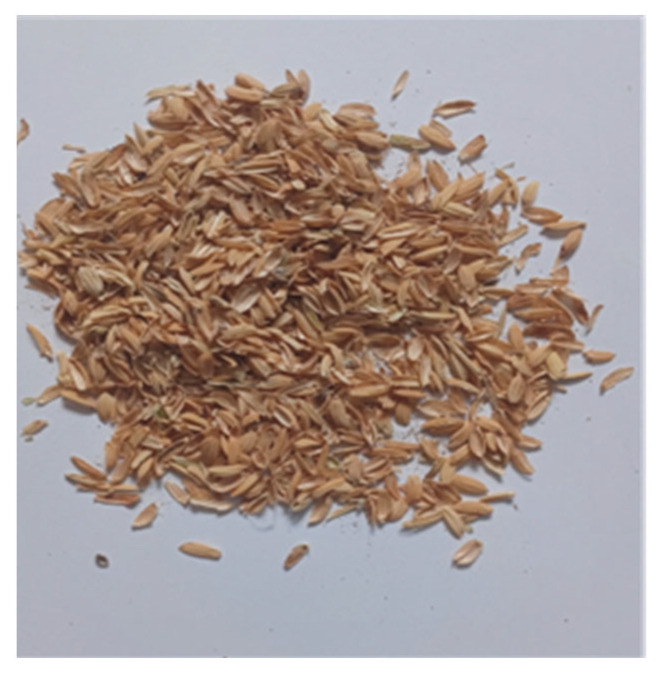
The appearance of rice husk.

By embedding RH in natural and/or synthetic binders and adding adhesives, in different combinations of mixing ratios, a range of twelve PRHs were produced under laboratory conditions (temperature of 23 ± 2 °C; relative air humidity of 50 ± 10%) at the INCERC Bucharest Branch Laboratory of NIRD URBAN-INCERC, from Bucharest, Romania. RHs, resulting from the food industry, were purchased from a local rice processing factory. As Chen et al. also report in his research [27], with a maximum length of 10 mm, a width of 2.3 mm, and a hard texture due to its microstructure, RHs have an appearance that is illustrated in Figure 1.

The binders were selected from the materials available on the Romanian construction materials market. Based on preliminary tests that analyzed ten binders with varying non-volatile matter content below 50%, it was determined that they do not provide good incorporation of the plant material. Therefore, the following were selected, as they allow good incorporation of RH through manual mixing:

- Bone glue granules (BGGs), with a density of 750 kg/m^3^, precursor material of the natural binder which was subsequently prepared in the laboratory. The preparation of the natural binder was achieved by soaking the granules in water (1:1) for approx. 12 h, then by heating the obtained gel in a Bain-Marie, up to a temperature of 60–70 °C, resulting in the bone glue (BG) in liquid phase.
- The synthetic binder (SB) was used in the form of three variants of finishing products; each of them, depending on their composition, was recommended for outdoor use, namely binder A, based on acrylic copolymer resins, and binder B, based on silicone resin, or for indoors, in environments with high humidity, as binder C, based on acrylic resin with biocide content. The three synthetic binders were water-based paints characterized by densities of 1.59 g/cm^3^, 1.54 g/cm^3^, and 1.44 g/cm^3^, respectively, according to Standard SREN ISO 2811-1 [64] and also by a non-volatile-matter content of 63.76%, 61.53%, and 53.12%, respectively, according to Standard SREN ISO 3251 [65]. The three synthetic products were selected as binders based on these criteria, on the one hand, considering them components having ecological characteristics, with low VOC emissions, and on the other hand, to also give the PRH an esthetic, finishing character.
- Two specific synthetic adhesives for wood, type D4, for exterior use were also selected, namely a polyvinyl acetate-based adhesive (AD1) and a polyurethane-based adhesive (AD2).

The bulk density for RH and also for BGG was determined by assimilating the Standard SREN 1097-3 [66], obtaining the values of 126.86 kg/m^3^ for the husks, respectively, of 750 kg/m^3^ for the granules.

All the tests above mentioned were carried out using standardized methods, under laboratory conditions (temperature of 23 ± 2 °C; relative air humidity of 50 ± 10%), with each quantified indicator determined as the arithmetic mean of three individual measurements, which ensured conditions of repeatability and reproducibility, with a measurement uncertainty of a maximum of 5%.

### 2.2. Mixtures for PRH

Twelve biocomposite mixtures were obtained, resulting in nine plasters containing rice husks and a synthetic binder (PRH-SBs), namely A1–A3, B1–B3, C1–C3, and three plasters having the natural bone glue binder (PRH-BG), namely A4, B4, and C4. The composition of the plasters expressed in mass ratios is presented in Table 1.

The determination of the optimal binder-to-RH mass ratios was carried out through successive trial-and-error attempts, using workability and the absence of cracks in the plaster upon drying as control indicators, as well as a minimal binder addition for proper application on the substrate. The motivation behind conditioning the binder quantity to be as low as possible is due to the following two elements:

- Maximizing the incorporation of as much RH as possible;
- The binder, by its nature, contributes to TVOC emissions, which is desired to be as low as possible. Therefore, reducing the binder quantity leads to a decrease in TVOC concentrations.

### 2.3. Sample Preparation

#### 2.3.1. Sample Preparation for Testing Thickness and Adhesion to the Concrete Substrate

**Figure 2:**
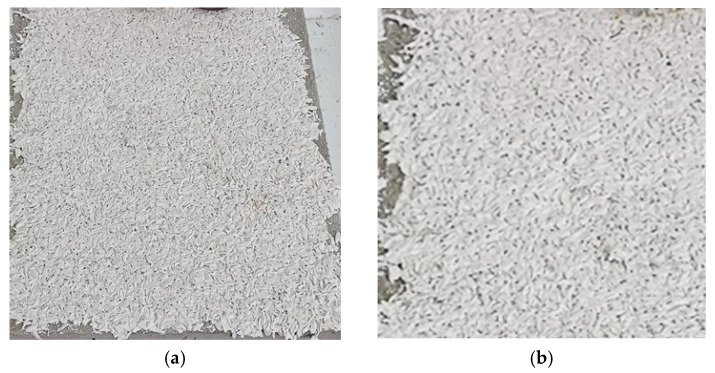
Appearance of the plaster A1 applied on the surface of a concrete slab: (**a**) overview of the A1 plaster; (**b**) detail of the A1 plaster (4×).

Each of the nine PRH-SBs was applied after priming the concrete surface according to the application instructions of each finishing product used as the synthetic binder in the plaster. In order to determine their thickness and adhesion to the concrete surface, the plasters were applied manually, with a stainless-steel trowel, in a single layer, on concrete slab surface measuring 500 × 500 mm^2^. Figure 2 illustrates the appearance of the plaster A1, as an overview (Figure 2a) and as a detail (Figure 2b), with the other PRH-SBs being visually similar.

**Figure 3:**
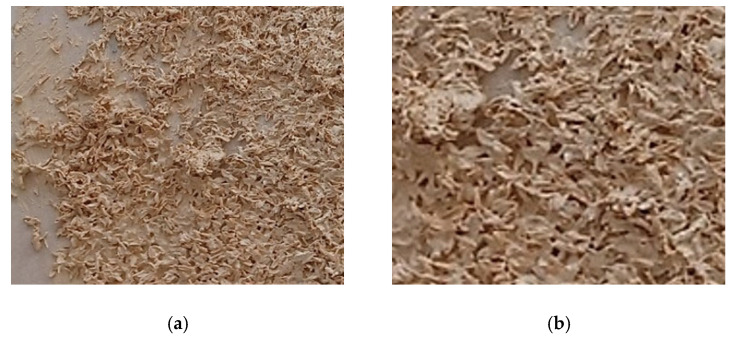
The appearance of a PRH-BG applied on a concrete surface. (**a**) Overview of the PRH-BG; (**b**) detail of the PRH-BG (2×).

During the laboratory work, a special situation was observed in the case of applying bone glue-bonded biocomposites to concrete surfaces. Introduced in various combinations in the designed recipes, together with one of the three synthetic binders, BG led to obtaining compatible mixtures, with good application on concrete surfaces, but with a relatively limited application time. This situation was found when applying the mixtures on relatively large-size concrete surfaces, of 50 × 50 cm^2^, which is a consequence of the way the BG must be prepared in order to get in a fluid state. There is a low possibility of keeping the mixture sufficiently long at a temperature at which, after homogenization, the biomaterial can be applied effectively on areas larger or at least equal to 2500 cm^2^. Due to this limitation, for three PRH-BGs, the application of biomaterials to the concrete slab was possible only as long as the BG had a temperature between 50 and 60 °C; there was insufficient time for a correct application over the entire 2500 cm^2^ surface of the sample. Once the temperature of the natural binder, implicitly of the biomaterial, began to fall below the specified range, its workability became increasingly precarious, generating an uneven coverage, with discontinuities, as can be observed in Figure 3, as an overview (Figure 3a) and as a detail (Figure 3b).

Following the aforementioned findings, the three variants of PRH-BG, namely A4, B4, and C4, were not applied on concrete surfaces anymore.

#### 2.3.2. Sample Preparation for Testing Thermal Insulation Properties

The thicknesses of the PRH-SBs and PRH-BGs are relatively small compared to the thicknesses of traditional materials used in construction, with thermal insulation characteristics, which made it impossible to directly measure the thermal resistance. Additionally, a particularity of the tested plasters is that they are applied as a support for practical use; they cannot be used as a stand-alone material. As a result, it was necessary to use a gypsum cardboard support layer characterized by low water vapor absorption, with a sufficiently large thickness (nominal thickness 12.50 mm).

Each of the twelve biomaterials was applied to both surfaces of twelve plasterboard boards (G1–G12) with nominal size of 300 × 300 × 12.5 mm. Unlike the situations encountered when the PRH-BGs were applied to concrete surfaces, the application of PRH-SBs and PRH-BGs to plasterboard surfaces was carried out without any difficulty.

**Figure 4:**
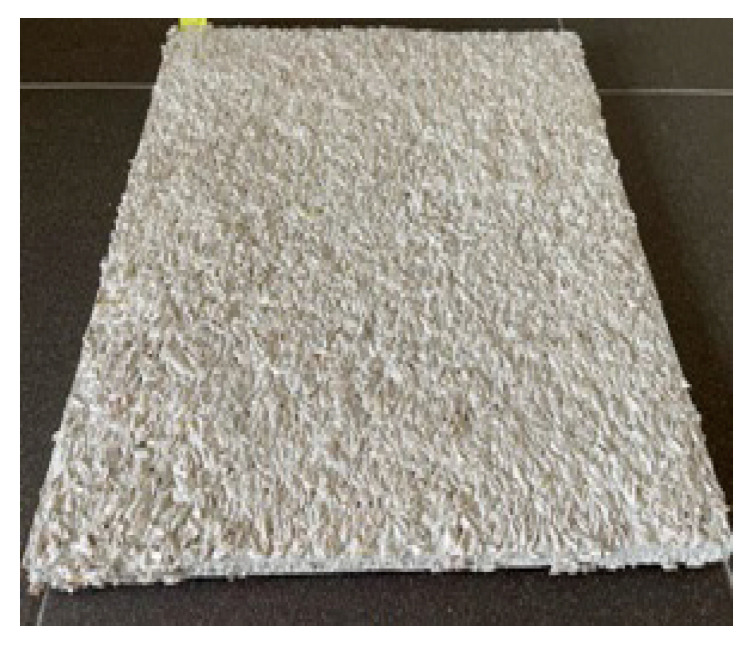
The appearance of a PRH applied on a plasterboard.

During the preparation of this set of samples, it was found that under the same environmental conditions during application, the nature and area of the support surface are decisive elements for a normal application of PRH. This statement is supported by the fact that, unlike the application of PRH-BGs on 2500 cm^2^ concrete surfaces, the execution of the work did not present any problems when applying the same biomaterials on 900 cm^2^ plasterboard support surfaces. Specifying that each of the three synthetic binders was white in color, for example, Figure 4 shows the appearance of a PRH applied to a gypsum board with dimensions of 300 × 300 mm^2^.

#### 2.3.3. Sample Preparation for Monitoring TVOC Emissions

Taking into account the compositions of PRH-SBs plasters, it was considered that the synthetic binder is one of the main sources of TVOC emissions, together with the synthetic adhesive. Among the plasters developed with each of the three synthetic binders, A, B, and C, only those with the acrylic binder C (PRH-C) were selected to be studied in terms of TVOC emissions. The selection was based on two criteria—first, the fact that in this product group, these plasters had the highest synthetic binder content, and second, it was considered that, due to its composition, binder C with biocide content would have the most significant TVOC emission; relevant results in this regard were also obtained in other studies conducted on decorative plasters containing sunflower shells [65]. Based on the above arguments, three plasters were studied in terms of TVOC emissions, namely C1, C2, and C3, the recipes being presented in Table 1, together with a control sample (CS), consisting exclusively of the C binder. Each of the three PRH-C test products were applied in one layer, with a stainless-steel trowel, to gypsum cardboard support surfaces measuring 50 × 50 × 12.5 mm. The CS was obtained by applying the C binder in two layers, by brushing on one side of the gypsum board, with no prior dilution. Four hours after the application of the second layer, the side on which the C product was not applied as well as all the edges of the support plate were covered with an aluminum foil, resulting in an emission surface of 0.2213 m^2^. The same operation was made for C1, C2, and C3, after applying the single layer of each biomaterial.

### 2.4. Experimental Testing Methods for the PRH

#### 2.4.1. Thickness and Adhesion to the Concrete Surface of the PRH-SBs

The nine PRH-SBs were characterized in terms of thickness and adhesion to the concrete substrate according to [63], after drying, hardening, and conditioning for seven days at (23 ± 2) °C and (50 ± 10)% relative humidity. The thickness was measured with an electronic caliper (YATO, accuracy of 0.02 mm). Adhesion was determined using the pull-off method (MATEST, Treviolo, Italy; accuracy and repeatability ± 1%).

#### 2.4.2. Characterization of the PRH-GBs and PRH-SBs in Terms of Thermal Insulation Properties

**Figure 5:**
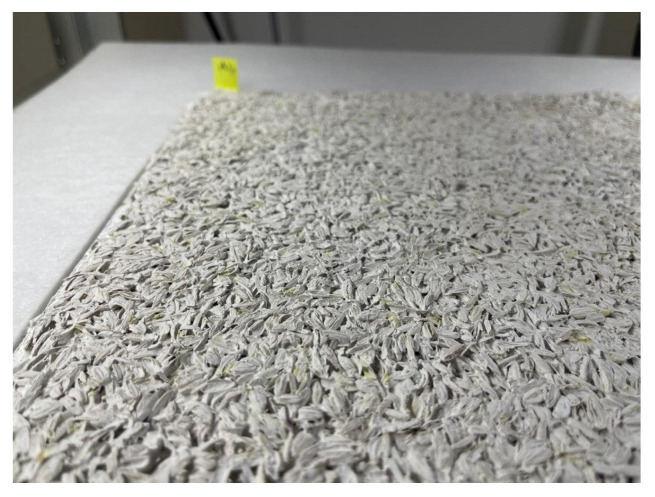
The sample with the tested PRH, placed in the thermal conductivity determination equipment with a perimeter, thermally insulating frame made of recycled PE non-woven material, in order to reduce thermal bridges.

The twelve plasters were characterized in terms of thermal insulation properties according to the Standard SR EN 12667 [67] standard, by the hot guarded plate method (λ-Meter EP500e, Lambda-Meßtechnik GmbH, Dresden, Germany); tests were carried out at the INCERC Bucharest Branch Laboratory of NIRD URBAN-INCERC. The working method is based on completing the following steps:

- Taking support samples from plasterboard and coding them for subsequent identification; conditioning of support samples at a temperature of 40 °C;
- Determining the thermal resistance and thermal conductivity of each sample, under stationary thermal regime conditions. Given the dimensions of the samples, a thermal insulation ring made of recycled PE non-woven material is used during testing to reduce thermal transfer in the guard area of the equipment (Figure 5);
- Application of PRH-SB or PRH-BG;
- Maturation of the coating for seven days through chemical reactions;
- Conditioning of the samples under conditions similar to those imposed on the support samples;
- Determination of the thermal resistance and thermal conductivity of each sample formed by the support plate and the tested plaster under stationary thermal regime.

The selection of plasterboard as the substrate for thermal conductivity measurements was based on the following two primary considerations: (1) the plasters are designed as surface-finishing materials that require a substrate in actual building applications and cannot function as standalone products, and (2) plasterboard represents a common substrate material in residential and commercial construction. The use of plasterboard with low water vapor absorption and sufficient thickness (12.5 mm) ensured dimensional stability during testing and minimized edge effects.

The differential measurement approach employed in this study effectively accounts for the substrate’s influence on thermal measurements. By measuring the thermal resistance of the bare plasterboard (R_G_) separately from the composite system (R_VG_), the thermal contribution of the plaster alone (R_V_) was isolated through subtraction (Equation (1)). This methodology assumes that thermal contact resistance at the plaster–plasterboard interface is minimal under steady-state conditions, which is reasonable given that (i) the plaster was applied in its intended manner as described in Section 2.3.2, ensuring good physical contact with the substrate; (ii) the samples underwent a seven-day curing period, allowing complete adhesion development; and (iii) the hot guarded plate method under stationary thermal regime minimizes transient interface effects.

Any residual interface effects present in our measurements would also exist in real-world applications, making our results representative of in-service thermal performance. The consistency of thermal conductivity values across different formulations (0.072–0.083 W/m·K) and their alignment with published data on similar rice husk-based materials [42,43,68] further validates the reliability of this measurement approach. Nevertheless, we acknowledge that complete isolation of interface thermal resistance remains an inherent limitation of testing thin coating materials that require substrate application.

The following thermal properties of the tested plasters were determined:

- Thermal resistance, using the following calculation relationship, appropriate for the stationary heat transfer regime:

(1) $$R_{V}=R_{VG}-R_{G}\;\left[\frac{m^{2}K}{W}\right]$$

where $R_{V}$—thermal resistance of plaster $\left[\frac{m^{2}K}{W}\right]$; $R_{VG}$—thermal resistance of the gypsum plasterboard specimen covered on both sides with the plaster to be tested $\;\left[\frac{m^{2}K}{W}\right]$; and $R_{G}$—thermal resistance of the plasterboard specimen $\;\left[\frac{m^{2}K}{W}\right]$.

- Thermal conductivity:

(2) $$\lambda_{V}=\frac{\delta_{V}}{R_{V}}\;[\frac{W}{m\cdot K}],$$

where $\lambda_{V}$—thermal conductivity of plaster $\left[\frac{W}{m\cdot K}\right],{\;\delta }_{V}$—thickness of plaster layers [*m*]; and$\;R_{V}$—thermal resistance of plaster $\left[\frac{m^{2}K}{W}\right]$.

This methodology is consistent with recent experimental protocols employed for rice husk-based thermal insulation characterization, including ASTM C-177 testing procedures validated in multiple contemporary studies [42,43,68].

#### 2.4.3. Volatile Organic Compound Emissions of the PRH-Cs

**Figure 6:**
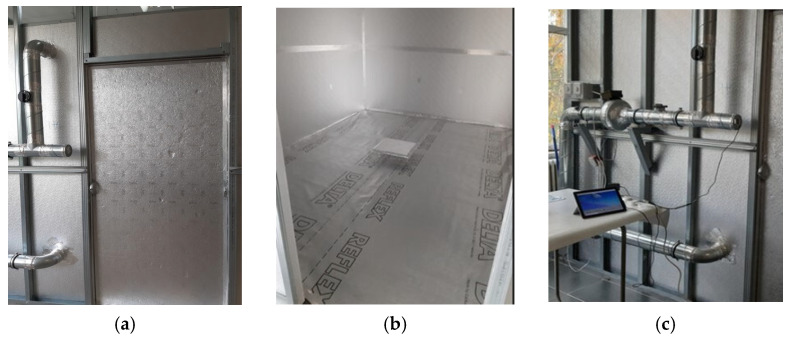
(**a**) The front side of S2 experimental stand for emission monitoring; (**b**) sample with the plaster to be tested, placed inside of the experimental stand S2; and (**c**) Direct Sense IQ-610 equipment for VOCs concentrations recording and a part of ventilation system.

Each of the three samples of PRH-C and the CS were subjected to emission monitoring in the S2 experimental stand, designed and implemented at the INCERC Bucharest Branch Laboratory, illustrated in Figure 6a,b (described in [69]).

The emission monitoring in the aforementioned stand was performed according to a monitoring protocol, in air recirculation mode [69]. The measurement of VOC emissions was carried out using Direct Sense IQ-610 equipment (Figure 6c) (GrayWolf Sensing Solutions, Shelton, CT, USA) [70,71,72], whose operating principle is based on electronic detection via a photo-ionization detector (PID) sensor. The measurement range is 20–20,000 ppb, with a resolution of 1 ppb, and the sampling interval for the VOC concentrations was one minute, with the total recording period of 24 h.

The equipment was calibrated before the measurements.

During the TVOC concentrations monitoring, the average temperature value was 20 ± 2 °C. The average relative humidity value was 55 ± 5%.

## 3. Results and Discussion

### 3.1. Characterization of the PRH-SBs in Terms of Thickness and Adhesion to the Concrete Surface

The thickness and the adhesion to the concrete surface of the nine PRH-SBs were determined; after seven days of application, the average values obtained being presented in Table 2.

Analyzing the experimental results obtained for the nine PRH-SBs applied on the concrete surface, it is observed that the average thickness values are between 2.71 mm and 6.26 mm, and for seven of the nine plasters, the adhesion exceeds the minimum value of 0.3 N/mm^2^, provided by the specification EN 15824—Specifications for exterior and interior plasters based on organic binders. Comparing the average adhesion values for each group of the three plasters (A3, B3, and C3) related to each synthetic binder, it is observed that the adhesion to concrete was higher in the cases where the biomaterial composition contained the polyurethane adhesive AD2, although it was used in smaller quantities than the polyvinyl acetate-based adhesive AD1.

It is important to point out that all the twelve PRH recipes were developed based on two hypotheses, namely the following:

- This type of plaster can be performed using synthetic binder available on the construction materials market as an interior or exterior finish. Considering the fact that binders with a minimum of 50% non-volatile content were used and that preliminary tests eliminated binders with non-volatile matter content lower than 50% due to poor RH incorporation capacity, it is considered that for the development of such a plaster, the appropriate binder should have a minimum non-volatile substance limit, this limit needing to be validated through additional tests. Therefore, in each recipe, the quantities of the corresponding components are RH-binder = 1:(min 5, max 8), noting that the differences consist in the amount of incorporated SB necessary to obtain a mixture characterized by good workability and application on concrete. Thus, it can be observed that the type of binder (acrylic copolymer, silicone, or acrylic) has a major influence regarding the possibility of compositional formulation of RH-based plasters.
- PRHs could be considered as decorative plasters that can be performed and applied also by individual users, the raw materials being accessible, and the technical equipment being minimal. The biomaterials are able to be mixed and homogenized manually or mechanically, until homogeneous and workable enough to allow manual application with a stainless-steel trowel. In this regard, in Figure 2, Figure 4 and Figure 5 it can be seen that, both when applying PRH-SBs on a concrete substrate and when applying PRH-BGs on plasterboard, a continuous, uniform layer resulted, without cracks visible to the naked eye upon drying, for the compositions prepared in the laboratory by simple manual mixing and application with a stainless-steel trowel.

Analyzing the results in Table 2 from the perspective of the first hypothesis specified above, taking into account the non-volatile-matter contents of the three synthetic binders (mentioned in Section 2.1), it can be seen that, for seven of the nine plasters, it was verified that this type of products can be made by using interior or exterior wall finishing paint, respecting the main condition that SB has a non-volatile-matter content of min. 50%. The fact that this contributes significantly to ensuring proper adhesion of the plaster to concrete was better observed in the case of the products A1 and A2 when, incorporating the binder A in smaller quantities than in product A3, with a smaller quantity of adhesives AD1, led to lower average adhesion values. It is also observed that plaster A3 has the highest adhesion among all nine products applied to concrete. Given that all products in the A3, B3, and C3 series, with AD1 content, had the same recipe, it could be considered that the difference in adhesion is due to a significant extent to the characteristics of the binder, more precisely its non-volatile-matter content.

The experimental results obtained at this stage indicate the potential for use in construction of seven of the nine PRH-SBs, as a result of their adequate adhesion to the concrete support surface. Moreover, the rustic texture of the products applied and the appearance given by the embedded SB binder led to the idea that the seven products could be considered, at this stage of the research, as decorative plasters applicable to this type of surface. It is also possible that the results obtained support the second hypothesis previously stated, i.e., that PRH-type plasters could be made and applied also by individual users. At this stage, the possibility of creating a pre-dosed multi-component product (plant material, binder, and possibly an additive) that can be marketed is anticipated, also representing a sustainable and accessible option.

Recent studies on bio-based plaster adhesion mechanisms have emphasized the importance of substrate-plaster interface chemistry. Research on clay-composite plasters incorporating industrial waste has shown that hydraulic lime as a partial clay substitute significantly improves adhesion to concrete substrates [73], while investigations on cement mortar adhesion revealed that roughcast application enhances bonding through hydrogen bond transfer mechanisms from mortar to concrete, resulting in homogeneous adhesion compared to heterogeneous bonding when mortar is directly applied [74]. The incorporation of polyurethane adhesive (AD2) in the PRH formulations (plasters A3, B3, and C3) consistently yielded higher adhesion values compared to formulations with polyvinyl acetate (AD1) or no additional adhesive, demonstrating the effectiveness of synthetic adhesive supplementation in natural fiber-based systems. This finding aligns with recent work on graphene-based additives in earth plasters, which showed improved strength and water resistance through enhanced interfacial bonding [75].

### 3.2. Characterization of the PRH-GB and PRH-SB in Terms of Thermal Insulation Properties

Table 3 presents the values for the thicknesses of the base plates G1 ÷ G12 (plates that are just the support, on which the studied plasters were applied), the thicknesses of each (support plate + applied PRH) ensemble, the average PRH thickness on one side of each tested sample, the thermal resistance R and the equivalent thermal conductivity of the tested PRH (values determined for the average sample temperature of 10 °C), as well as some clarifications and observations on test results.

**Figure 7:**
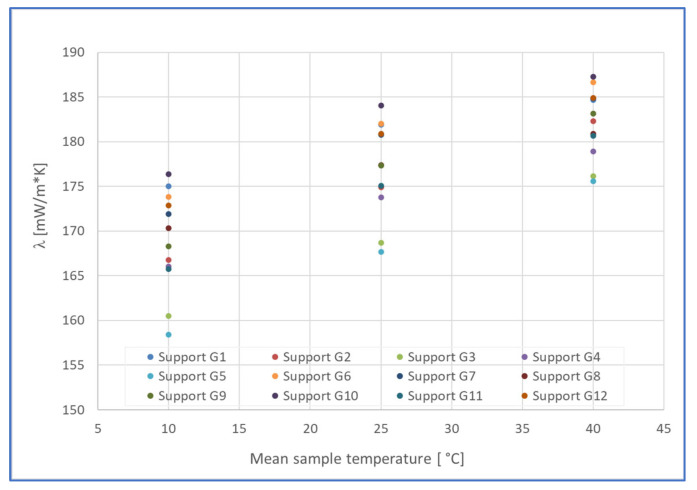
Thermal conductivity of gypsum board support plates [mW/m·K], in the range 10–40 °C.

**Figure 8:**
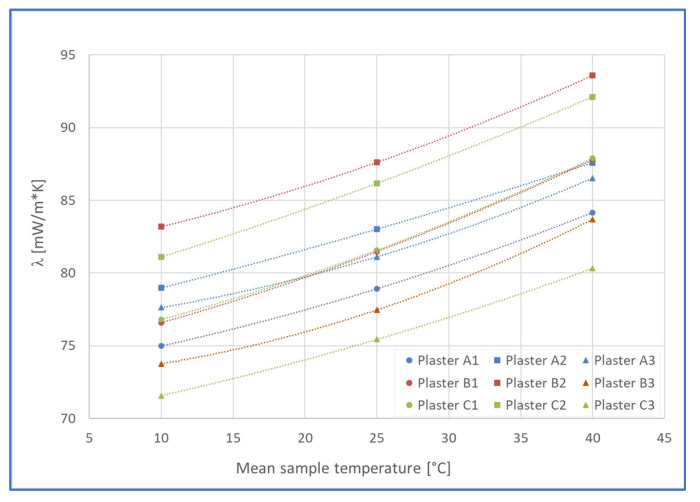
Thermal conductivity of the tested PRH [mW/m·K], in the range 10–40 °C.

Figure 7 and Figure 8 present a centralized graphical representation of the experimental results obtained regarding the variation in the thermal conductivity values of the twelve gypsum board support plates before the application of PRHs, in the temperature range 10–40 °C, respectively; the variation in the thermal conductivity of the twelve PRHs was applied to the gypsum board in the same temperature range.

It is mentioned that since the PTH-BGs could not be applied to concrete surfaces, their testing was to be carried out in terms of thermotechnical characteristics, because in the case of this determination, the innovative biocomponent products had to be applied to plasterboard, according to the test method. However, in the first seven days after the application of PRH-BGs on the plasterboard, during the maturation period of the samples, they were affected by a partial cleavage phenomenon, most likely as a result of the action that the hot biomaterials had on the plasterboard upon application. In the case of the respective products, namely A4, B4, and C4, the obtained results were not taken into account, even though they were the best (Table 3) samples considered to be in an inadequate state to provide correct results.

Referring to the “partial cleavage” phenomenon visually observed after the maturation period of the samples, we could consider that this is mainly the result of the action of thermal stress manifested on the gypsum board during the application of PRH-BGs, when the temperature of the biomaterials was about 50–60 °C. In addition, the occurrence of the physical or/and mechanical degradation of the gypsum boards mentioned above may have been caused by both the humidity of the biomaterials during the application stage and the physical–chemical interactions between the natural binder BG and the gypsum board. These interactions could have occurred both during the drying and hardening period of the plaster, in the first 7 days after application, and during the conditioning period of the samples before testing.

The phenomenon intensified and continued over time. So, approximately two months after the determination of the thermal insulation properties, during which the samples were kept under standard laboratory conditions (temperature of 23 ± 2 °C; relative air humidity of 50 ± 10%), the boards covered with PRH-BGs were increasingly affected both by the alteration of the cohesiveness of the board core and by the detachment of the cardboard layer on which the plasters were applied.

**Figure 9:**
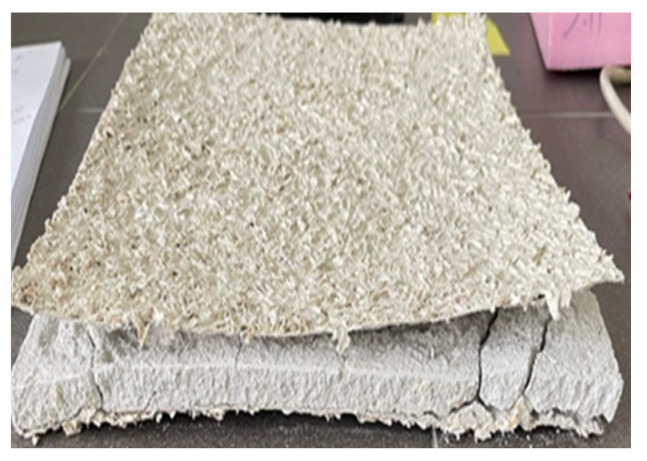
Evolution of the initial cleavage phenomenon of the samples.

In the case of PRH-BGs applied on gypsum cardboard, the evolution of the initial cleavage phenomenon of the samples (Figure 9) demonstrates the evident deficiencies of applying this type of plasters on such support surfaces, clearly affecting the behavior of PRH-BGs even when they are exposed to standard laboratory environmental conditions.

From the analysis of the values obtained from the tests carried out to determine the thermal protection properties of plasters in the PRH-SB category, the following results emerge:

1. Each of the tested samples presents characteristics specific to thermal protection materials, although the layer thicknesses are much smaller compared to those of traditional materials;
2. Considering the evolution of the thermal conductivity coefficient during the testing interval, it follows that the tested materials are relatively homogeneous and without air inclusions;
3. The best value recorded for thermal conductivity is that of the A3 coating, namely 71.57 mW/m·K, with this sample being also relatively constant in the test temperature range.

Except for the unfavorable evolution of the PRH-BG samples in the experimental tests performed, Table 4 presents the results obtained for the seven PRH-SB plasters in terms of rice husks content in total mixture, thickness on plasterboard surfaces, and thermal conductivity.

Analyzing the recipes designed for PRHs and the experimental results obtained in terms of their average values of RH content, thickness when the plasters are applied on the plasterboard and thermal insulation properties, the following aspects can be highlighted:

1. The best results were obtained for PRH-SB, the ones applicable to both concrete and plasterboard surfaces;
2. The thickness of the seven PRH-SBs is between 4.20 mm and 7.70 mm when applied to plasterboard. It is worth noting that the two best thermal conductivity values were obtained for plasters B3 and C3, which had the highest average thicknesses when applied to gypsum board and contained AD2;
3. In terms of RH content, for the seven PRH-SBs with adhesion above 0.3 N/mm^2^, it varied between 10.64% and 13.63%, the former being characteristic for C3, the PRH-SB with the best thermal conductivity;
4. Taking into account the obtained results, it could be assumed that a PRH-SB can be made using synthetic binder available on the construction materials market as an interior or exterior finish, provided that it is characterized by a non-volatile-matter content of min. 50%. At the same time, it could be considered that any of the PRH-SB could be made and applied even by individual users, being a two-in-one product, as a decorative plaster and also as a plaster having characteristics specific to thermal protection materials. Considering the experimental results obtained in this stage of the research regarding the characteristics of PRHs in terms of application, adhesion to concrete and plasterboard, thermal insulation properties, and impact on indoor air quality, the use of this type of plasters would have a series of limitations concerning mainly application and usage conditions than do-it-yourself (DIY) applicability. The difficulties when applying PRH-BGs on concrete surfaces and the initial partial cleavage, followed by the significant deteriorations of the samples when applied on plasterboards, indicated that this kind of plaster is not recommended to be applied on those two substrates. Based on the results of this research, the PRH-SBs could have DIY applicability on both concrete and drywall surfaces and could be recommended for use in partially covered outdoor spaces.

From studying the specialized literature, regarding the use of rice husks in construction, the general approach consists of partially replacing the base material of the traditional product with this natural by-product [24,39,76]. For example, the study [38] presents the effects of incorporating RH in different proportions, between 7% and 20% in an ordinary plaster, the beneficial effects being materialized both in reductions in the plaster density from 1244.2 kg/m^3^ to 1055.4 kg/m^3^ but especially in the decrease in its thermal conductivity from 0.94 W/m·K to 0.67 W/m·K.

Comparing [39] with the approach of the present research, a central similarity can be observed regarding the correlations between the % of RH added and the thermal conductivity value of the resulting plaster. Thus, if in the research by Manatura and colleagues [38], with additions of 7%, 15%, and 20%RH resulted in plasters with thermal conductivities of 0.83 W/m·K, 0.72 W/m·K, and 0.67 W/m·K, respectively, in the present paper, for RH contents of 10.64%, plasters with thermal conductivities of 0.071 W/m·K (plaster C3) and 0.073 W/m·K (plaster B3) were obtained. Therefore, the increase in RH content up to 13.63% lead to a slightly increased thermal conductivity, for example, of 0.081 W/m·K (plaster C2).

In fact, with the increase in % RH, there is a tendency for a slight variation in thermal conductivity, ascending or descending, considering that with the increase in RH content, upon application, the natural material has a tendency to arrange itself so as to occupy as much of the free spaces in the porous structure of the plaster as possible. Therefore, 10.64% could be considered an optimal RH content of the plaster (variant C3), in terms of both resulting thickness (6.57 mm) and thermal conductivity, the adhesion to concrete (0.5 N/mm^2^) also being higher than the limit value of 0.3 N/mm^2^ required by [63].

The thermal conductivity values obtained in this study (0.072–0.083 W/m·K for PRH-SB) align remarkably well with recent experimental investigations on rice husk-based thermal insulation materials. Specifically, Cigarruista Solís et al. [42] reported a thermal conductivity of 0.073 W/m·K, which is nearly identical to the best-performing C3 plaster (0.072 W/m·K) developed in the present work. This strong agreement validates both the experimental approach and the material composition employed. Studies on other bio-based thermal insulation materials have reported comparable ranges as follows: Posidonia oceanica-based composites achieved 0.052–0.067 W/m·K [77], while recycled paper with natural fibers reached 0.027 W/m·K [78], cellulose-biochar cryogels demonstrated 0.041 W/m·K [79], and sheep wool/hemp mixtures attained 0.033–0.043 W/m·K [80]. A comprehensive analysis of natural fiber-derived thermal insulation materials by Lian et al. [81] established a typical range of 0.03–0.10 W/m·K, with the observation that convective heat transfer and thermal radiation become dominant in low-density structures below 50 kg/m^3^. The present PRH plasters, with thermal conductivity in the mid-range of bio-based materials, represent a practical balance between insulation performance and structural applicability, particularly considering their thin-layer application (2.71–6.26 mm on concrete; 4.20–7.70 mm on plasterboard) compared to traditional bulk insulation materials.

There are also significant differences between the two approaches. First, in the two different categories of plasters studied, namely a classic plaster in which the RH partially replaced the initial material [24,39,76], respectively, a PRH, a plaster made predominantly from RH, together with a synthetic binder, with or without a synthetic adhesive. A second important difference is the state of the RH when it is embedded in the plaster matrix in two situations. If ref. [39] used RH after preliminary stages of drying at 100 °C and sorting so that only 800—micron particles were used, in the present research, RH was used in its natural state and dimensions, without any additional drying, an aspect that favorably influences the costs associated with this type of plaster.

Regarding the determination of the thermal conductivity, in the present research, the tests were carried out using the hot guarded plate method, as in other experimental studies carried out on building materials with thermal insulation characteristics containing plant materials [24,59]. However, testing these products required a specific approach due to their relatively small thicknesses compared to those of traditional materials with thermal insulation characteristics used in construction. In addition, a particularity of the tested plasters was that, since they could not be used as an independent material, they were applied on a plasterboard support, with a sufficiently large thickness of 12.50 mm. It was also found that the nature and area of the support surface are decisive elements for a normal application of PRH-BGs, due to the limitations regarding the preparation method of the bone glue binder, the temperature of the mixture upon application, but also the actual application time on surfaces of approximately 2500 cm^2^. Another particular aspect encountered during this type of testing was the finding that during the maturation time of the PHR-BG samples, they degraded through a slight cleavage of the plasterboard support, most likely due to the temperature of approximately 50 °C of the applied BG-based biomaterials, which practically nullified the relevance of the results obtained for the respective samples.

### 3.3. Characterization of the PRH-C in Terms of Volatile Organic Compound Emissions

**Figure 10:**
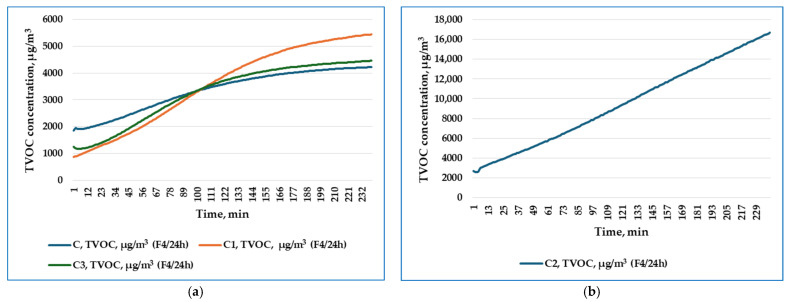
Variation over time (first four hours of monitoring) of TVOC concentration for (**a**) C, C1, and C3, tested products; (**b**) C2 product.

**Figure 11:**
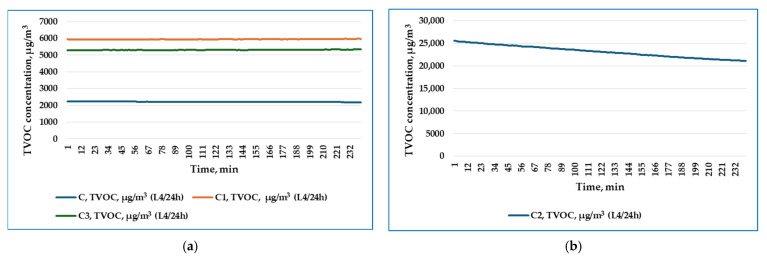
Variation over time (last four hours of monitoring) of TVOC concentration for (**a**) C, C1, and C3, tested products; (**b**) C2 product.

The variation over time of the TVOC concentration emitted by the tested plasters C1, C2, and C3, and the control sample C, in the first four hours of monitoring (F4/24 h), in air recirculation mode, are graphically represented in Figure 10a,b, respectively, while those in the last four hours of monitoring (L4/24 h) are represented in Figure 11a,b.

The average values of the TVOC concentration emitted by the tested plasters C1, C2, and C3, and the control sample C, in air recirculation mode, in the first four hours of monitoring, respectively, in the last four hours of monitoring, are presented in Table 5.

From the point of view of the average values of TVOC concentration, in the first four hours of monitoring, the descending series for the four variants is C2 > C1 > C > C3. In the last four hours of monitoring, the descending series of average TVOC concentration values is C2 > C1 > C3 > C.

Based on the experimental results obtained regarding the TVOC emissions of the three plasters and of the control sample, the following main aspects can be observed:

- Through the determined values, the monitoring highlights clear compositional differences between the control sample—the binder containing biocides—and the C2 plaster—whose composition includes both that of the control sample and that of the AD2 adhesive. It is also necessary to take into account the fact that, within the formulation, the presence of the adhesive (AD1 or AD2) determines the difference in TVOC emissions in the first 4 h of emission/monitoring.
- Regarding the three plasters tested, it was found that at the same rice husk content, in order to obtain comparable workability, the decrease in the amount of binder was compensated by incorporating a quantity of AD1 or AD2 adhesive, with AD2 > AD1, in which case it can be considered that the AD2 adhesive became the one that determined the difference in emissions from a quantitative point of view. However, it is suggested that, by extending monitoring over longer periods of time, during different stages of maturation of each coating, for at least 7 days after application, the information would be more relevant regarding the total VOC emissions characteristic of dry PRH-SBs, put into operation.
- This research aimed to study the impact that the binder of PRHs has on indoor air quality. Due to its synthetic nature and biocide content, PRH-C was chosen to be studied in this research because it was considered that the binder C has the potential to emit the highest levels of TVOCs. It is intended that, in a future stage of the research related to PRHs, to expand the study by using other types of binders.

Regarding studying the impact on indoor air quality by monitoring TVOC emissions of PRH-SBs, the specialized literature [59] states that the larger the particle size in the coating structure, the greater the surface area of the coating that comes into contact with the air, which contributes to higher emissions in short-term measurements. This is also the case for PRH, especially due to the relatively large size of rice husks, but also due to the much greater thickness of a plaster than a paint, this information explains the high values of TVOC emissions recorded during the 24 h of monitoring and also highlights the need that for PRH to study VOC emissions for at least 8 days after application.

However, in the literature, the contribution of some products commonly used for the substrate, such as plaster, does not appear sufficiently investigated, although they are an integral part of the final solution. Few studies are therefore available, such as Kwok et al. [82], showing the influence of substrate on VOCs emissions from paint and varnishes and finding that VOCs emission is closely related to the substrate type.

The TVOC emission values recorded in this study (3272–9470 µg/m^3^ within 24 h) are consistent with baseline emission levels reported for conventional building materials, where unoptimized formulations typically yield TVOC concentrations of approximately 3775 µg/m^3^ [62]. Recent research on bio-based material emissions has demonstrated that environmental conditions significantly influence VOC release rates, with elevated temperature and humidity consistently increasing emissions, while higher air exchange rates reduce indoor VOC accumulation [60]. Long-term emission characterization studies spanning 431 days have established that while initial VOC concentrations (C_0_) diminish exponentially with material aging, diffusion coefficients (Dₘ) and partition coefficients (K) remain relatively stable [61], suggesting that the high initial emissions observed in the present 24 h monitoring period would decrease substantially over subsequent days. Importantly, studies comparing conventional and bio-based formulations have shown that careful selection of natural binders can reduce TVOC emissions by 70–80% (from ~3775 µg/m^3^ to ~880 µg/m^3^) [62], indicating significant potential for optimization of PRH formulations in future work. Similarly, incorporation of bio-based residues such as perinic acids into PLA and PCL polymer matrices applied to wood-based surfaces has demonstrated enhanced barrier properties, significantly reducing both TVOC and formaldehyde emissions [83].

Natural building materials have also demonstrated superior adsorptive capacities for VOCs compared to conventional materials [84], suggesting that rice husk-based plasters may not only emit fewer VOCs when optimized but could also actively contribute to improved indoor air quality through pollutant adsorption. Recent work on adaptive ventilation control systems has further demonstrated that proper environmental management strategies can significantly reduce indoor VOC concentrations while maintaining energy efficiency [85], highlighting the importance of holistic approaches to indoor air quality improvement.

Accordingly, an extension of the monitoring period of TVOC emissions up to eight or even 28 days after the application of the plasters would allow a relevant assessment of these construction products from the point of view of their impact on the quality of the indoor environment. Further research directions on PRH-SB will consist of studying their durability and acoustic properties.

From a circular economy perspective, the successful development of PRH plasters aligns with global trends in agro-industrial waste valorization for construction applications. Recent systematic reviews analyzing the 2017–2024 literature have documented increasing applications of agricultural by-products across multiple industries, with construction materials representing a particularly promising valorization pathway that minimizes environmental impact while generating economic value [86]. Studies specifically examining manufacturing processes using agricultural waste have demonstrated that such materials can achieve established building standards while contributing to economic, environmental, and social sustainability goals [87]. The use of rice husks in their natural state, without additional drying or processing as employed in this work, further enhances the circular economy benefits by minimizing energy consumption during material preparation. Previous research by the authors has demonstrated the potential of innovative thermal insulation products derived from waste materials to contribute to circular economy objectives [88], establishing a foundation for the present investigation of rice husk-based plasters.

Similar approach aligns with broader European and international initiatives promoting resource efficiency and waste reduction in the construction sector. For example, Thailand’s Bio-Circular-Green economic framework that aims to optimize of flood sediment by the addition of rice husk and sodium bentonite obtaining adobe bricks [44], or treating soils polluted with heavy metals by using washing methods with solutions based on natural precursors, such as humic acid; therefore, combining the benefit of decontamination has the advantage of biocompatibility [89], or through eco-friendly bioleaching procedures—bacterial leaching [90]. In an even broader sense, the increasing importance of green products and procedures can reach remarkable levels, such as, for example, the creation of biopolymer membranes with energy applications, a significant step towards green energy storage devices [91] or the application of the cross-linking method using riboflavin, known as vitamin B2, as a photoinitiator in tissue engineering [92].

## 4. Conclusions

The central goal of this work was to identify a new possible direction for the use in construction of a natural agro-industrial by-product resulting from the food industry, namely the rice husks.

This paper presented the materials and the compositional aspects of the obtained plasters, and the criteria and the methods used for the interdisciplinary study of the resulting products. The thickness and adhesion to the concrete surfaces, the thermal insulation properties, and the impact on indoor air quality by TVOC emissions monitoring were the quantitative indicators that were determined for characterizing the plasters. The good and very good experimental results obtained are also presented.

The element of originality lies in obtaining plasters by incorporating rice husks into various binders and adhesives, unlike most of the studies reported in the specialized literature, which aimed at partially replacing the binder in a traditional product with rice husks.

The two hypotheses on which the experimental research focused on developing the PRHs were

1. This type of plaster can be performed using mainly RH and any synthetic binder available on the construction materials market as an interior or exterior finish, provided that it has a particular non-volatile-matter content;
2. PRH type plasters could be performed and applied also by individual users, due to the accessibility of the raw material, without the need for any prior preparation, and because of the minimal technical equipment required.

Twelve PRHs were obtained, of which nine PHR-SBs and three PHR-BGs. Due to the application limitations generated by the conditions of preparation and use of the natural binder BG, the research was continued only on the nine PHR-SBs, in order to characterize and establish their field of use, these being studied in terms of thickness when applied to concrete surfaces, adhesion to concrete, thermal conductivity and impact on indoor air quality, by monitoring TVOC emissions.

Of the nine plasters studied, seven were characterized by average thickness values ranging between 2.71 mm and 6.26 mm when applied to concrete surfaces and adhesions to concrete ranging between 0.33 N/mm^2^ and 0.65 N/mm^2^, the adherence obtained values corresponding to a plaster based on organic binders, according to EN 15824.

These seven PRH-SBs also have thermal insulation characteristics, their thermal conductivity being between 0.071 W/m·K and 0.083 W/m·K, even if when applied to plasterboard surfaces, they have thicknesses ranging between 4.20 mm and 7.70 mm, small values compared to those of traditional thermal insulation products. These thermal performance values align well with recent experimental investigations on rice husk thermal insulators (0.073 W/m·K) [42] and fall within the established range for bio-based thermal insulation materials (0.03–0.1 W/m·K) [81], confirming the viability of rice husk plasters as a thermal protection solution.

In terms of the impact that the three PRH-SBs studied have on indoor air quality, TVOC emissions indicated high values both in the first four hours and in the last four hours of the 24 h monitoring period, due to the large surfaces of the PRH-SBs in contact with the air, this type of plaster having a porous structure.

Taking into account the obtained results, it could be considered that the first hypothesis was confirmed, meaning that PRH-SB can be made using synthetic binder available on the construction materials market as an interior or exterior finish, provided that it is characterized by a non-volatile-matter content of min. 50%.

The second hypothesis, meaning PRH-SB could be made and applied even by individual users, being a two-in-one product, as a decorative plaster and also as a plaster having characteristics specific to thermal protection materials, was also confirmed. Considering the experimental results obtained in this stage of the research regarding the characteristics of PRHs, the use of this type of plasters would have a series of limitations concerning mainly application and usage conditions than DIY applicability. Based on the results of this research, the PRH-SBs could have DIY applicability on both concrete and plasterboard surfaces in partially covered outdoor spaces.

At the present stage of the research on plasters obtained by incorporating rice husks, it is considered that further tests are necessary to fully evaluate their potential for use in construction. It is appreciated that the favorable interaction between the support surface and the PRH-SBs, as well as the experimental results obtained, constitutes premises for good durability of these plasters when applied to concrete and exposed in partially covered outdoor spaces. It is intended that the durability aspects of the PRH-SB, such as testing of moisture resistance, freeze–thaw stability, and long-term adhesion, will be addressed in future research.

The findings confirmed that the use of rice husks for obtaining PRH represents a direction of valorization with an encouraging potential. The results indicate very good thermal performance and good adherence values, though comprehensive characterization is ongoing.

## Figures and Tables

**Table 1:** Characterization of the plasters (mass ratio).

Plasters	RH	A	B	C	BG	AD1	AD2
A1	1	5.58	-	-	-	-	-
A2	1	5.83	-	-	-	0.50	-
A3	1	7	-	-	-	-	1.40
A4	1	5	-	-	5	0.60	-
B1	1	-	6.70	-	-	-	-
B2	1	-	7	-	-	1	-
B3	1	-	7	-	-	-	1.40
B4	1	-	5	-	5.70	0.60	-
C1	1	-	-	8	-	-	-
C2	1	-	-	7	-	0.80	-
C3	1	-	-	7	-	-	1.40
C4	1	-	-	5	5	0.60	-

**Table 2:** Average values of the thickness and adhesion of PRH-SB applied on the concrete support surface and of the thickness of PRH-SB applied on plasterboard surface.

PRH-SB	A1	A2	A3	B1	B2	B3	C1	C2	C3
Average values of the thickness of PRH-SB applied on concrete surface (mm)	4.35	3.87	4.78	3.76	4.57	6.26	5.61	2.71	4.02
Average values of the adhesion of PRH-SB to concrete surface (N/mm^2^)	0.23	0.18	0.65	0.34	0.33	0.41	0.44	0.48	0.50
Average values of the thickness of PRH-SB applied on plasterboard surface (mm)	-	-	5.97	4.53	4.52	7.70	5.11	4.20	6.57

**Table 3:** Results of the measurements performed to determine the thermal insulation properties of the PRH-SB and PRH-GB tested plasters.

Sample Name	Thickness of the Support, [mm]	Average of PRH Thickness on One Side of the Tested Sample, [mm]	Thermal Resistance R, [(m^2^K)/W]	Equivalent Thermal Conductivity of the Tested PRH, [mW/m·K]	Observations
Plasterboard support G1	12.59		0.0719		Support plate
Plasterboard support G2	12.7		0.0762		Support plate
Plasterboard support G3	12.69		0.0791		Support plate
Plasterboard support G4	12.7		0.0765		Support plate
Plasterboard support G5	12.82		0.0809		Support plate
Plasterboard support G6	12.7		0.0731		Support plate
Plasterboard support G7	12.59		0.0732		Support plate
Plasterboard support G8	12.63		0.0742		Support plate
Plasterboard support G9	12.63		0.0751		Support plate
Plasterboard support G10	12.68		0.0719		Support plate
Plasterboard support G11	12.69		0.0766		Support plate
Plasterboard support G12	12.76		0.0738		Support plate
Plasterboard support G1+ Tested plaster C1	22.82	5.11	0.2051	76.80	
Plasterboard support G2+ Tested plaster C2	21.11	4.20	0.1799	81.10	
Plasterboard support G3+ Tested plaster C3	25.83	6.57	0.2627	71.57	Minimum thermal conductivity of the tested sample
Plasterboard support G4+ Tested plaster A1	23.22	5.26	0.2168	74.98	
Plasterboard support G5+ Tested plaster A2	21.57	4.37	0.1917	78.97	
Plasterboard support G6+ Tested plaster A3	24.64	5.97	0.2269	77.63	
Plasterboard support G7+ Tested plaster A4	28.9	8.15	0.3231	65.27	Partial cleavage of the support specimen
Plasterboard support G8+ Tested plaster C4	33.21	10.29	0.4014	62.90	Partial cleavage of the support specimen
Plasterboard support G9+ Tested plaster B4	25.43	6.40	0.2718	65.04	Partial cleavage of the support specimen
Plasterboard support G10+ Tested plaster B1	21.74	4.53	0.1902	76.58	
Plasterboard support G11+ Tested plaster B2	21.74	4.52	0.1854	83.18	Maximum thermal conductivity of the tested sample
Plasterboard support G12+ Tested plaster B3	28.16	7.70	0.2826	73.75	

**Table 4:** Results obtained for the seven PRH-SB in terms of thickness on concrete surfaces, adhesion to concrete, thickness on plasterboard surfaces, and thermal conductivity.

PRH-SB	RH Content, [%] in Total Mixture	Average Values of the Thickness of PRH-SB Applied on Plasterboard Support, [mm]	Equivalent Thermal Conductivity of the Tested PRH, [mW/m·K]
A3	10.64	5.97	77.63
B1	12.98	4.53	76.58
B2	11.11	4.52	83.18
B3	10.64	7.70	73.75
C1	76.80	5.11	76.80
C2	81.10	4.20	81.10
C3	51.57	6.57	71.57

**Table 5:** Average values of the TVOC concentration for studied plasters and control sample.

Type of Plaster/ Average Values of TVOC Concentration [µg/m^3^]	C	C1	C2	C3
In the first 4 h of monitoring	3342	3547	9470	3272
In the last 4 h of monitoring	2210	5940	23,175	5305

## Data Availability

The original contributions presented in this study are included in the article. Further inquiries can be directed to the corresponding author.
